# Pharmacopuncture for Cancer Care: A Systematic Review

**DOI:** 10.1155/2014/804746

**Published:** 2014-05-12

**Authors:** Soyeon Cheon, Xiuyu Zhang, In-Seon Lee, Seung-Hun Cho, Younbyoung Chae, Hyangsook Lee

**Affiliations:** ^1^Department of Korean Medical Science, Graduate School, Kyung Hee University, Kyung Hee Dae-ro 26, Dongdaemun-gu, Seoul 130-701, Republic of Korea; ^2^Acupuncture and Meridian Science Research Center, College of Korean Medicine, Kyung Hee University, Kyung Hee Dae-ro 26, Dongdaemun-gu, Seoul 130-701, Republic of Korea; ^3^Hospital of Korean Medicine, Kyung Hee University Medical Center, Kyung Hee Dae-ro 23, Dongdaemun-gu, Seoul 130-701, Republic of Korea

## Abstract

*Background*. Pharmacopuncture, injection to acupoints with pharmacological medication or herbal medicine, is a new acupuncture therapy widely available in Korea and China for cancer-related symptoms. However, the evidence is yet to be clear. *Objective*. To determine pharmacopuncture's effectiveness on cancer-related symptoms. *Methods*. Eleven databases were searched for randomized controlled trials of pharmacopuncture in cancer patients. The Cochrane risk of bias (ROB) assessment tool was used for quality assessment. *Results*. Twenty-two studies involving 2,459 patients were included. Five trials of chemotherapy-induced nausea and vomiting (CINV) underwent meta-analysis. Pharmacopuncture significantly relieved severity of CINV compared with control group (3 trials, risk ratio (RR) 1.28, 95% confidence interval (CI) = 1.14–1.44). The frequency of CINV was also significantly reduced with pharmacopuncture (2 trials, RR 2.47, 95% CI = 2.12–2.89). Seventeen trials studied various symptoms, and in most studies, pharmacopuncture significantly relieved pain, ileus, hiccup, fever, and gastrointestinal symptoms and improved quality of life in various cancer patients. ROB was generally high. *Conclusion*. It may be suggested with caution that pharmacopuncture may help various symptom relief in cancer patients, but it is hard to draw a firm conclusion due to clinical heterogeneity and high ROB of the included studies, hence warranting further investigation.

## 1. Introduction

Cancer is a well-known health problem worldwide. In 2008, GLOBOCAN [1] estimated about 12.7 million cancer cases worldwide, and a recent study made estimates of more than 1.6 million new cases in USA alone for 2013 [2]. More recent report from World Health Organization (WHO) is estimating that global incidence of cancer will rise up to 22 million per year within the next two decades [3]. Although there have been significant advances in the field of cancer treatments in the past decades [4, 5], effective management of cancer and its related symptoms still leaves much to be desired, and moreover, adverse events (AEs) that come along in the course of cancer treatment are another problem.

The frequently experienced AEs associated with cancer treatment, such as pain, nausea and vomiting, fatigue, or constipation, lead patients and researchers to seek new approaches, and among various options available, there is complementary and alternative medicine (CAM) as one approach [6, 7]. Among various CAM therapies, pharmacopuncture, also known as acupoint injection, herbal acupuncture, aqua acupuncture, or aquapuncture, that is, pharmacological medication or purified herbal medicine injected to acupoints, is a new acupuncture therapy that combines acupuncture therapy and medication. It is now widely used in China and Korea for a range of symptoms including cancer-related symptoms [8], and an Australian study reported that it induces higher de-qi sensation compared to traditional acupuncture, which may be an indication that pharmacopuncture could provide stronger clinical response than traditional acupuncture [9]. Also, some studies reported that pharmacopuncture in addition to manual acupuncture produced better clinical outcomes, such as significant improvement in pain and function for patients with herniated intervertebral disc [10, 11], or significantly less pain and shorter duration of pain for patients with postauricular pain from peripheral facial paralysis [12], compared with manual acupuncture alone. Although evidence for its efficacy is piling up [13–15], no systematic reviews for assessing pharmacopuncture for cancer care have been published yet.

Therefore, the aim of this review was to critically summarize and assess the current evidence from randomized controlled trials (RCTs) that investigated pharmacopuncture's effectiveness on cancer-related symptoms and to help clinicians and patients make informed decision making.

## 2. Methods

### 2.1. Data Sources and Searches

We searched for RCTs and systematic reviews of pharmacopuncture in cancer care. Electronic searches were conducted in PubMed, Embase, the Cochrane Central Register of Controlled Trials (CENTRAL), Cumulative Index to Nursing and Allied Health Literature (CINAHL), Chinese National Knowledge Infrastructure (CNKI), and related Korean databases including KoreaMed, KMbase, Riss4U, KISS, OASIS, and DBPIA from inception through March 2013. Also, trial registries (http://www.controlled-trials.com/, http://www.clinicaltrials.gov/) were searched to find any relevant ongoing or unpublished trials. No language restriction was imposed. Search strategy used for PubMed was as follows: ((pharmacopuncture*[All Fields] OR “herbal acupuncture” [All Fields] OR “aqua acupuncture” [All Fields] OR aquapuncture*[All Fields] OR “acupoint injection” [All Fields]) AND (Neoplasms [MeSH] OR Neoplasms*[TI] OR Cancer*[TI] OR Tumor*[TI] OR Tumour*[TI] OR Carcinoma [MeSH] OR Carcinoma*[TI] OR Adenocarcinoma [MeSH] OR Adenocarcinoma*[TI] OR adenomatous [TI] OR Lymphoma [MeSH] OR lymphom*[TI] OR lymphedema*[TI] OR Sarcoma [MeSH] OR Sarcoma*[TI] OR “Antineoplastic agents” [MeSH] OR antineoplas*[TI] OR ((adenom*[TI] OR adenopath*[TI]) AND malignant*[TI]))) AND ((“Meta-Analysis as Topic” [Mesh] OR “Meta-Analysis” [PT] OR (meta [TIAB] AND analys*[TIAB]) OR metaanalys*[TIAB] OR (systematic [TIAB] AND (review*[TIAB] OR overview*[TIAB])) OR “Review Literature as Topic” [Mesh] OR Cochrane [TIAB] OR embase [TIAB] OR psychlit [TIAB] OR psyclit [TIAB] OR psychinfo [TIAB] OR psycinfo [TIAB] OR cinahl [TIAB] OR cinhal [TIAB] OR “Science citation index” [TIAB] OR bias [TIAB] cancerlit [TIAB] (reference [TIAB] AND list*[TIAB]) OR bibliograph*[TIAB] OR (hand [TIAB] AND search*[TIAB]) OR “relevant journals” [TIAB] OR (manual [TIAB] AND search*[TIAB]) OR ((“selection criteria” [TIAB] OR “data extraction” [TIAB]) AND review [PT]) NOT (Comment [PT] OR Letter [PT] OR Editorial [PT] OR (Animals [MeSH] NOT (Animals [MeSH] AND Human [MeSH])))) OR (“randomized controlled trial” [PT] OR “controlled clinical trial” [PT] OR random*[TIAB] OR placebo [TIAB] OR “drug therapy” [Subheading] OR trial [TIAB] OR groups [TIAB] NOT (Animals [MeSH] NOT Humans [MeSH]))). For individual search of each database, slight modifications were applied to the above strategy.

### 2.2. Study Selection

Search results were screened by one author (SC) based on the title and the abstract first and then were selected for the final analysis by two authors (SC and XZ). To be included in the systematic review, studies should involve and randomly allocate cancer patients to a pharmacopuncture group or a control group. If there are other cointerventions such as chemotherapy, radiotherapy, and/or other palliative or supportive care, they should be given identically to both groups. Studies were included if they reported clinical symptom improvement, that is, studies reporting laboratory findings only were excluded.

### 2.3. Data Extraction and Assessment of Risk of Bias (ROB)

The following information was subject to collecting data: first author's name, country, year of publication, number of participants, types of cancer, details of interventions for experimental and control groups, cointerventions, outcome measures, results for the outcome measures, and reported AEs associated with pharmacopuncture.

ROB was assessed independently by two authors (SC and I-SL) using the modified ROB assessment tool from the Cochrane Handbook [39]. The criteria consist of 6 items that might be related to selection bias (random sequence generation and allocation concealment), performance bias (blinding of participants and personnel), detection bias (blinding of outcome assessment), attrition bias (incomplete outcome data), and reporting bias (selective outcome reporting). Any discrepancies between the two reviewers were resolved by a thorough discussion with a corresponding author (HL) until consensus was reached.

### 2.4. Data Analysis

Studies were combined in the analysis according to the outcome measure, intervention type, and/or control type. When there was more than one control groups within a study, we chose the control group that administered the identical drug that was used for the intervention group over the other control group that used different type of drug, because that would make the comparison between the two groups limited to the efficacy of injection type only, for example, pharmacopuncture or intravenous (IV) injection. Data were pooled using a random effects model. The impact of pharmacopuncture on dichotomous outcomes was expressed with risk ratio (RR) with 95% confidence intervals (CI) and the effect of pharmacopuncture on continuous data was expressed with mean difference (MD) with 95% CI. To generate forest plots of pooled estimates with 95% CI, Review Manager (Version 5.2 Copenhagen: The Nordic Cochrane Centre, The Cochrane Collaboration, 2012) was used. Visual inspection of forest plots and a chi-squared test with a *P* value less than 0.1 were used to assess heterogeneity among studies. The *I* ^2^ test was also used to quantify inconsistencies among the pooled studies and if important heterogeneity existed, we explored the reasons for heterogeneity. The *I* ^2^ statistic indicates the proportion of variability among studies that is not explained by chance alone and we considered that an *I* ^2^ value of 50% or more indicated a substantial level of heterogeneity [40, 41]. When data pooling was deemed misleading due to clinical heterogeneity and a small number of studies, a qualitative synthesis was undertaken.

## 3. Results

### 3.1. Study Selection and Description

**Figure 1:**
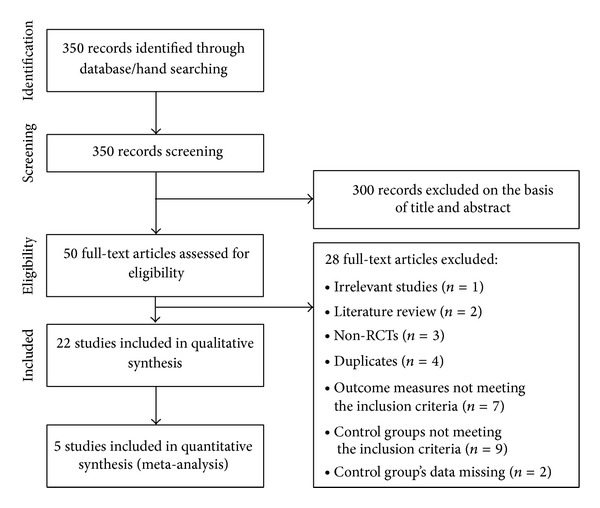
Flow diagram for selection of studies. RCTs: randomized controlled trials.

A total of 350 articles were initially identified using our search strategy. Three-hundred articles were excluded based on the title and abstract, leaving 50 articles to be screened with full text. Of these, 22 studies involving 2,459 participants met all the inclusion criteria and were included in our review (Figure 1). All 22 studies originated from China, and among them, one study [26] was published in English, and the others were all in Chinese. One article [18] was a doctoral dissertation and 21 were published in peer-reviewed journals. Seven studies recruited participants of certain cancer types such as late gastric cancer, ovarian cancer, hematologic cancer, rectal cancer, and upper gastrointestinal (GI) cancer [19, 24, 26, 27, 31, 32, 36]. The other 15 studies involved participants with various types of cancer [16–18, 20–23, 25, 28, 29, 33–35, 37, 38]. We could categorize the topics of the included studies under 5 individual symptoms, that is, pain (8 studies [16–23]), chemotherapy-induced nausea and vomiting (CINV, 6 studies [24–29]), ileus (two studies [31, 32]), hiccup (two studies [33, 34]), and fever (two studies [35, 36]). In addition, there were two other categories which were quality of life (QOL) [37] and GI symptoms in general [38]. For intervention, 4 studies [18, 23, 28, 37] used Chinese herbal medicine injection at the acupoints, and 18 studies used pharmacological medication injection. All studies compared pharmacopuncture with conventional medication and among them, 4 studies [19, 26, 27, 29] had two control groups. All 4 of them compared pharmacopuncture with non-pharmacopuncture of an identical drug that was used in the intervention group for one control; for another control group, two studies [19, 26] used manual acupuncture, and two others [27, 29] used injection of different pharmacological medication. Within the other 18 studies that had only one control group, while all of them used pharmacological medication, 7 studies used identical drug that was used in the intervention group and 11 studies used different kind of drugs.

Characteristics of 8 studies of pain are summarized in Table 1. Five studies [19–23] were of sample sizes over 100 and three studies [16–18] had a smaller size between 40 and 52. Six studies were on CINV and their characteristics are summarized in Table 2. Sample sizes ranged from 51 to 480 [24–29]. Various control groups were used: intramuscular (IM) injection or manual acupuncture (one study), IV injection (4 studies), and oral medication (one study). Characteristics of the other included studies for symptoms of ileus, hiccup, fever, QOL, and GI symptoms are tabulated in Table 3. Three studies for ileus and QOL had a sample size between 108 and 160 [31, 32, 37], while the other 5 studies' sample size varied from 38 to 58 [33–36, 38]. Study designs were pharmacopuncture versus IM injection (5 studies), pharmacopuncture versus IM injection or oral intake (one study), pharmacopuncture combined with routine postoperative therapy versus routine postoperative therapy alone (one study), and pharmacopuncture with routine chemotherapy versus IV injection with routine chemotherapy.

### 3.2. ROB Assessment

All of the included articles had a high ROB, lacking proper blinding measures for participants, personnel, and outcome assessors which were participants themselves in all the cases. Only one study reported how they concealed allocation by using sequentially numbered opaque envelopes [26], and none of the other studies reported either how sequence was generated or how concealment of allocation was done so the ROB was evaluated to be unclear. Twenty-one studies had a low ROB for incomplete outcome data and selective outcome reporting categories, but one study [36] had a high ROB for the latter because it reported positive results of symptoms that were not predefined in its method (Table 4).

### 3.3. Outcomes of the Included Studies by Symptom

#### 3.3.1. Pain

All 8 studies used responder rate to evaluate pain (Table 1). They were qualitatively analyzed due to clinical and statistical heterogeneity. Responder rates in 4 studies [17–19, 22] were calculated by percentage of number of participants with any improvement out of total number of participants. Shen [18] also looked at onset time and duration of analgesia in addition to responder rate. In other three studies [20, 21, 23], responder rate was defined as the proportion of number of participants with improvement from the intervention and no sleep disturbance. One study [16] used percentage of number of participants with improvement and no need of analgesia out of total number of participants.

In these 8 studies, only one study [17] reported that there was no significant difference of responder rate between pharmacopuncture and control group, and 7 others favored pharmacopuncture. Among the latter 7 studies, three studies had only one control group with one outcome measure, and their details and results are as follows. Liu [16] compared 100 mg of bucinnazine pharmacopuncture at ST36 with bucinnazine IM injection. De-qi was elicited by needle lifting/inserting and twisting. Bai [22] injected 20 mg of nefopam to ST36 and compared responder rate with that of 50 mg pethidine IM injection. Guan et al. [23] compared distilled and purified Chinese herb (chansu, naoyanghua, chuanwu, banxia, xionghuang, and bingpian) pharmacopuncture with western medicine. Acupoints for pharmacopuncture were selected according to each participant's cancer type; for example, participants with lung cancer received pharmacopuncture at LU1, LU2, BL13, and BL17. The above three studies' results showed that pharmacopuncture performs significantly (*P* < 0.05) better than the control.

The other 4 studies had more complicated design than the previous three studies, that is, more than one control group, outcome measure, or outcome measurement time. One study [19] that had two control groups reported that pethidine injection at acupoint ST36 had significantly (*P* < 0.01) better analgesic effects than manual acupuncture at ST36 or pethidine IM injection with flexible dosage between 50 and 100 mg. Another study [18] that had two outcome measures compared 0.1–0.3 mL of Stauntoniae injection at ear acupoints with oral intake of 5–15 mg oxycodone according to degree of pain. While there was no significant difference for responder rate between the two groups (*P* > 0.05), the pharmacopuncture group demonstrated faster onset time (mean ± SD, 17.7 ± 6.9 min versus 31.4 ± 6.6 min; *P* < 0.01) and longer duration (18.2 ± 1.3 min versus 11.8 ± 1.0 min; *P* < 0.01) of analgesia compared to control group. In the study of Wang et al. [20], outcome was measured at more than one time-point. In the pharmacopuncture group, anisodamine hydrochloride 10 mg, dexamethasone 5 mg, and energy synbiotics 4 mL were injected at bilateral ST36, and 500 *μ*g of vitamin B12 was injected at bilateral SP10. The control group received IM injection of 100 mg pethidine. Injections were given once a day for both groups, and participants were asked about the pain right after the injection, 30 minutes after the injection, and 2, 4, 6 hours after the injection, and then every two hours afterward except sleeping time. No significant difference (*P* > 0.05) was detected when they compared the results of two groups for 30 minutes to 4 hours. The other study [21] by the same authors was done upon a different group of participants, and intervention group and control group were identical. However, this study checked outcome measures 5 times: right after the injection and 4, 24, 72, and 96 hours later. Participants on pharmacopuncture treatment benefited at all time-points except at 4 hours after injection.

In the one study [17], pharmacopuncture group received bucinnazine injection at acupoint ST36, and the control group had IM injection, while 4.2 mg of fentanyl patch was given to both groups. There was no significant difference in the responder rate between the two groups.

#### 3.3.2. CINV

Six trials involving 1,150 participants tested pharmacopuncture with usual care for CINV. All studies favored pharmacopuncture over the control group, but outcome measures varied. Five out of six studies reported responder rate as an outcome measure. One study [24] used responder rate that was calculated by percentage of number of participants with any improvement from the intervention out of total number of participants. Two studies [25, 28] defined the participants with the WHO grades 0 and 1 as responder [30]. Two studies [27, 29] calculated responder rate using emesis episodes: number of participants with less than 4 emesis episodes per day [27] and less than three emesis episodes per day as a responder [29]. One study [26] used two outcome measures, that is, a total number of emesis episodes in 21 days and a proportion of emesis-free days in the same period.

You et al.'s study [26] with participants with ovarian cancer tested injection of 50 mg of vitamin B6 either at bilateral PC6 or intramuscularly. There was also a third group and they were treated with manual acupuncture at bilateral PC6. Pharmacopuncture and manual acupuncture were given once every other day with de-qi being elicited. In the control group, IM injection was given twice daily. Total number of emesis episodes and proportion of emesis-free days over 21 days were compared among three groups, and the pharmacopuncture group significantly reduced emesis compared to both control groups (*P* < 0.01).

**Figure 2:**
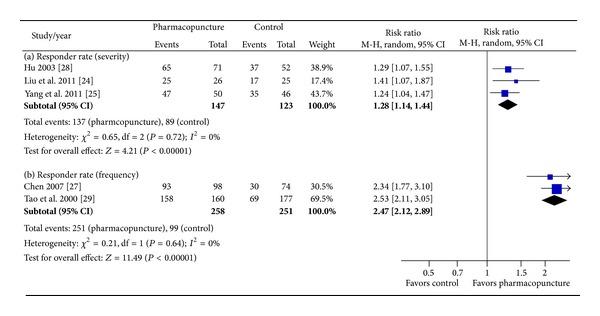
Forest plot of the effect of pharmacopuncture for CINV. CINV: chemotherapy-induced nausea and vomiting.

Among remaining 5 studies, three studies' responder rates were determined by severity of symptoms; Liu et al. [24] used 25 mg of promethazine for injection at bilateral ST36 for pharmacopuncture group. For control group, they used 10 mg metoclopramide IV injection. Pharmacopuncture group's responder rate was significantly higher than that of control group (96.2% versus 68%; *P* < 0.05). Yang et al. [25] also reported that significantly more participants in the pharmacopuncture group responded to the treatment than the control group (78% versus 52.1%; *P* < 0.05). The pharmacopuncture group was given 10 mg of metoclopramide at bilateral ST36 once daily. On the other hand, 5 mg of tropisetron IV injection was given to control group twice daily. Hu [28] injected 4 mL of Chinese herbs (huangqi or danggui) once a day for pharmacopuncture group at 5 acupoints: bilateral ST36, bilateral SP10, and BL23. Control group had oral medication of 50 mg of batilol, 20 mg of leucogen, and 20 mg vitamin B6, three times a day. Reported responder rate indicated that pharmacopuncture group had significantly (*P* < 0.01) better outcome than control group. Pooling these three studies yielded a significant benefit of pharmacopuncture against control group in symptom improvement of CINV (RR 1.28, 95% CI (1.14, 1.44), *χ* ^2^ = 0.65, df = 2, *P* = 0.72; *I* ^2^ = 0%; Figure 2(a)).

In the other two studies, number of emetic episodes was used to calculate responder rate. Chen [27] compared metoclopramide injections at acupoints with two control groups where IV injection of metoclopramide and IV injection of granisetron were given; pharmacopuncture group had 10 mg of injection for left PC6 and CV10 each, 15 minutes before chemotherapy, and then had another 10 mg each at right PC6 and CV10 right after chemotherapy. Metoclopramide control group had 20 mg injection each 15 minutes before and after chemotherapy, and granisetron group had 3 mg injection before chemotherapy. Results showed that pharmacopuncture had a higher responder rate than metoclopramide control group (95% versus 40.5%; *P* < 0.01), and no difference emerged when compared with granisetron group (95% versus 96.5%; *P* > 0.05). Tao et al. [29] also compared three groups. Pharmacopuncture group had 5 mg of metoclopramide and 1.25 mg of diazepam injection at bilateral PC6 and ST36, 30 minutes before chemotherapy, then 10 mg of metoclopramide right after chemotherapy. In a similar way, one control group had 20 mg of metoclopramide and 5 mg of diazepam and then 20 mg of metoclopramide, IV injection. The other group was given 8 mg of IV injection of ondansetron 30 minutes before and right after chemotherapy. Responder rate was reported for each group and pharmacopuncture group had better results than metoclopramide control group (98.8% versus 43.1%; *P* < 0.01) and no different result when compared with ondansetron group (98.8% versus 94.4%; *P* > 0.05). Combining these two studies showed that pharmacopuncture significantly reduced frequency of CINV compared with usual care (RR 2.47, 95% CI (2.12, 2.89), *χ* ^2^ = 0.21, df = 1, *P* = 0.64; *I* ^2^ = 0%; Figure 2(b)).

#### 3.3.3. Ileus, Hiccup, Fever, QOL, and GI Symptoms

Of the 8 studies included, 5 studies of fever [35, 36], hiccup [33, 34], and QOL [37] reported responder rate, that is, number of participants with any improvement from the intervention out of total number of participants (%). However, Xue [37] mentioned that they used Karnofsky score to assess improvement. Two studies [31, 32] of postoperative ileus measured time that the patient first passed a bowel movement. The last study [38] used WHO grades [30] to evaluate GI symptoms.

Chen et al. [31] and Feng et al. [32] studied effectiveness of pharmacopuncture for postoperative recovery of colorectal cancer patients. Rectal cancer patients participated in the study of Chen et al. [31] and the study reported that pharmacopuncture group's time that the patient first passed a bowel movement was significantly shorter than that of control group (45.1 ± 8.64 hours (h) versus 74.7 ± 16.32 h; *P* < 0.05). Pharmacopuncture group received injection of neostigmine at bilateral ST36, 0.5 mL each in addition to routine postoperative care twice daily, while the control group was given routine postoperative care alone. In Feng et al. study [32], colon cancer patients were recruited. Time that the patient first passed a bowel movement in the pharmacopuncture group was significantly shorter than that of the control group (29.6 ± 3.2 h versus 48.1 ± 5.3 h; *P* < 0.01). Both groups received 100 mg of vitamin B1 injection, but intervention group's injection was at bilateral ST36 with de-qi being elicited, and control group received IM injection.

Two studies [33, 34] had participants with hiccup. Sui and Zhang [33] reported that pharmacopuncture group had significantly higher responder rate than control group (*P* < 0.05). Intervention group was injected with 10 mg of anisodamine at ST36 twice a day. Control group got 10 mg of anisodamine injected intramuscularly twice a day. Xia et al. [34] also reported that intervention group had higher responder rate than control group, but the difference was not significant (*P* > 0.05). They injected 1.25 mg of atropine at bilateral ST36 and 10 mg each of vitamin B1 and B12 at bilateral BL17 for pharmacopuncture group. Control group had either oral intake of 0.3 mg of atropine three times a day, or 0.5 mg IM injection of atropine.

Two studies [35, 36] of fever drew a conclusion that pharmacopuncture group had significantly better result compared to control group (*P* < 0.05). In Yan et al. study [35], the intervention group had pharmacopuncture treatment with 50,000 *μ*/2 mL of recombinant human interleukin-2 (rIL-2) at ST36 once daily. For control group, rIL-2 (100,000 *μ*/2 mL) IV was administered once daily. Another study by the same author [36] used dexamethasone to compare pharmacopuncture and IM injection; pharmacopuncture group got 1 mL of dexamethasone at bilateral ST36 alternately once a day and de-qi was elicited before the injection. The control group had 10 mg of IM injection three times a day. Treatments continued for 5 days.

Xue [37] looked at participants' QOL and used Karnofsky score to calculate responder rate of the two groups. Pharmacopuncture significantly improved QOL compared to control group (*P* < 0.01). Intervention group had Huangqi injection at bilateral ST36 and the injection was once a day, alternately, with a dose of 2 mL. Control group received synbiotics IV injection once a day, and also lipid and albumin were given twice weekly. Treatment spanned two weeks.

Lastly, Zhao et al. [38] investigated the effect of metoclopramide pharmacopuncture on GI symptoms, that is, nausea, vomiting, diarrhea, and xerostomia. They reported that intervention group and control group did not have significantly different results (*P* > 0.05). In addition to routine chemotherapy, the intervention group received 10 mg injection at bilateral ST36 once a day, and the control group had IM injection. They assessed the improvement of GI symptoms on WHO 5 grades [30] starting from 0 to 5, grade 0 being the best outcome. From pharmacopuncture group, one participant matched criteria for grade 0. Then there were 6, 8, 0, and 0 participants for grades 1, 2, 3, and 4. In control group, none of the participants were suitable for grade 0, and 4 were in grade 1. Then there were 9, 2, and 0 participants for grades 2, 3, and 4.

### 3.4. Safety Evaluation

Of the 22 studies, 50% of the studies did not mention AEs at all, and the other half described AEs or mentioned that there were no AEs reported by the participants (Table 5). Five studies out of 11 reported that there were no AEs in the intervention group.

In the 6 studies that reported specific AEs for both groups, all mentioned that AEs were resolved after the end of treatment or spontaneously. However, none of the studies clarified causal relationship of AEs and pharmacopuncture. Six studies could be categorized into three groups: identical drug used for both intervention and control groups [16, 33, 36], different drugs used for each group [20, 21], and one study [29] with two control groups which correspond to identical drug and different drug each. While the other four studies provided numerical data by each group, two studies [20, 21] lacked such information and just listed patient-reported AEs.

Among the five studies that reported numerical data for intervention and control group, the incidence rate ranged from 0% to 36.0% for intervention group [18, 33] and 33.3% to 72.0% for control group [18, 36]. No dropout or withdrawal was reported in any of the studies.

## 4. Discussion

In this review, qualitative synthesis and partial meta-analysis regarding effectiveness of pharmacopuncture for cancer-related symptoms were done. Twenty-two included studies involving 2,459 participants were classified under 7 categories and for studies of CINV, a meta-analysis was performed. Pharmacopuncture significantly reduced severity and frequency of CINV compared with usual care. For studies that were under categories other than CINV, various study design and methods led us to qualitative synthesis of evidence. Among 8 studies under pain category, one study [17] reported a result indicating no difference between pharmacopuncture and conventional IM analgesic injection, but others were mostly in favor of pharmacopuncture against conventional treatments. In studies related to ileus, hiccup, fever, QOL, and GI symptoms, reported figures for results varied, but overall, all results were favoring pharmacopuncture. Fifty percent (11/22) of the included studies reported occurrence or absence of AEs, and among the reported AEs, there were no serious AEs that led to withdrawals from the study. However, we argue that the overall estimated results from either qualitative synthesis or meta-analysis should be interpreted with caution because of included studies' innate limitations, especially considering the fact that all included studies suffered from a high ROB and clinical heterogeneity.

We used Cochrane ROB criteria [39] for assessing ROB for the included studies. Overall ROB was high for the included studies; all failed to address the random sequence generation method in detail, and also all studies had unclear risk for allocation concealment except one [26] that mentioned usage of central randomization and sequentially numbered opaque envelopes. Participant blinding in pharmacopuncture studies may be more easily achievable than other nonpharmacological interventions such as acupuncture because pharmacopuncture is mostly compared against IV or IM injection. Nevertheless, none of the participants, personnel, and outcome assessors were blinded in the included studies and this could have brought performance and/or detection bias. High ROB of the primary studies in this field has been one of the major challenges for CAM to establish its place in evidence-based cancer care [42, 43]. Our review is no exception; the limitation of present study mainly lies on the high ROB of the included studies which was criticized above.

Another limitation of this review is from the clinical heterogeneity of the included studies. Study participants were often of different types and stages of cancer, causes of complained symptoms were not specified, duration of intervention and follow up length after the intervention were missing in some studies, and in many cases some of the selected control groups were not the best evidence-based treatment available [44, 45]. As with acupuncture, pharmacopuncture intervention varied greatly across trials that estimating its effectiveness under the single term of pharmacopuncture may be practically difficult. All these resulted in clinical heterogeneity of the included studies, which precluded us from pooling studies or drawing a definitive conclusion. Therefore, our findings should be interpreted with consideration of these limitations.

In RCTs, reporting harms as well as its benefits for an intervention is crucial to achieve a balanced perspective on the intervention. According to a guideline for better reporting of harms in RCTs, not only description of AEs for each arm should be provided, but also more rigor explanations like definitions for AEs or how harms-related information was collected should be reported [46]. In this review, only half of the studies briefly reported information regarding AEs, that is, just listing the symptoms, and no causality nor any further explanation was given. There were 5 studies reporting that there were no AEs, but this should be read cautiously because an intervention cannot be labeled safe merely based on the absence of information [47]. Interestingly, of the 4 studies that used identical drug for both groups, three studies' reported AEs from intervention group totally overlapped with those of control group. For example, Sui and Zhang [33] reported that there were a number of participants with xerostomia for both groups injected with anisodamine. Also, two other studies [20, 21] that administered anisodamine for their pharmacopuncture group reported AEs such as xerostomia along with hot flashes and blurred vision, which are all commonly known AEs of anisodamine itself [48, 49]. This can be suggesting that those AEs may be caused by the drug itself and not by the modality of pharmacopuncture. Still, more convincing evidence is needed to accurately assess the safety of pharmacopuncture.

One of the strengths of this systematic review is that we conducted comprehensive electronic database search and hand search for articles written in English, Chinese, and Korean. However, we cannot rule out that we might have missed some relevant articles. Moreover, all the included studies were from China at the end, so generalization of this review's finding is somewhat limited. Nonetheless, pharmacopuncture is evidently gaining popularity [8, 50], yet there was no other systematic review assessing pharmacopuncture's efficacy for various cancer-related symptoms. Therefore, this review serves as the first summarized evidence of its kind and may provide basis for further studies.

Pharmacopuncture has served as a novel and useful treatment for various diseases, usually in China and Korea [8, 50]. Given that most of its usage and relevant studies are from locally limited area, some consideration may be required before it is used worldwide, but the amount of data is increasing continuously [50] and so far the results are promising [51, 52]. For instance, a case series of bee venom pharmacopuncture for chemotherapy-induced peripheral neuropathy reported improvement of symptoms in patients and, importantly, there were no related AEs [52]. There was also a noticeable pilot study from University of California, San Francisco, that tested pharmacopuncture for primary dysmenorrhea, and they suggested that pharmacopuncture is highly acceptable among American women as well [51].

With a growing interest and increasing use of CAM in general, cancer patients are even more interested in various modalities of CAM, and they usually view it in a positive way with high levels of satisfaction [6, 53]. In this context, pharmacopuncture's utility for cancer-related symptoms is worth noticing. Especially, a brief Chinese review article of pharmacopuncture for cancer-related symptoms concluded that pharmacopuncture is not only effective but also cost-effective [54]. However, positive findings of that review can be consequences of publication bias and/or other methodological flaws. Also, the review itself suffers from poor reporting such as lacking proper explanation of which databases were searched with what kind of inclusion criteria. Though our review has a similar limitation, the execution of structured assessment and synthesis of up-to-date evidence may have been better delivered in our review to provide the best evidence possible.

This review suggests that the current evidence on pharmacopuncture for cancer-related symptoms is not sufficient yet. Included studies do report favorable results, but limitations from their quality make it hard to fully substantiate pharmacopuncture's effectiveness. To come up with a strong level of evidence, future studies should work on improving their quality and reporting. For instance, reporting part of the study can improve and become more reliable just by following existing guidelines. The Consolidated Standards of Reporting Trials (CONSORT) statement [55] is a well-recognized tool researchers can refer to. For trials reporting results of cancer treatment, there is also a classic guideline specifically for that purpose [56]. Referring to such guideline will improve quality of a study by giving detailed information about participants' status. Also, since practice of pharmacopuncture is very similar to acupuncture treatment in many steps, applying the Standards for Reporting Interventions in Clinical Trials of Acupuncture (STRICTA) [57] with suitable modification will be helpful as well. For example, 18 studies included in this review injected medication at ST36 but did not clarify why it was chosen. There was an interesting study from China that compared responder rate of injecting metoclopramide at different acupoints for CINV, and they reported that ST36 group showed significantly better result compared to LI4 and PC6 group (95.3% versus 79.1%, *P* < 0.05) [58]. Providing rationale or relevant evidence for chosen acupoints will improve the completeness and transparency of reporting in a study and add accuracy for future interpretation and replication of the study [57]. In this review, only handful of studies were subject to a meta-analysis due to high heterogeneity. When there are enough number of high-quality studies for each symptom, strength of evidence for pharmacopuncture may increase, and it will contribute to improvement of provided care for cancer patients.

## 5. Conclusion

We conclude that the level of evidence is not strong enough to draw any firm conclusion and gives us only a cautious suggestion that pharmacopuncture may help alleviate cancer-related pain, CINV, and other various symptoms such as ileus, hiccup, fever, QOL, and GI symptoms. To confirm this finding, further rigorously designed and conducted studies are required.

## Figures and Tables

**Table 1:** Characteristics of the included studies: pain.

Author	Sample size (M/F) Cancer type	Intervention (*n*)	Control (*n*)	Outcome measures	Result	
					Intervention	Control
Liu 2010 [16]	50 (31/19) Various	Bucinnazine (100 mg) injection at ST35 (*n* = 28)	Bucinnazine (100 mg), IM (*n* = 22)	Responder rate (%)^a^	85.7%*	59.1%
Li 2009 [17]	40 (22/18) Various	Fentanyl plaster (4.2 mg) and Bucinnazine injection at ST36 (*n* = 20) q8h	Fentanyl plaster (4.2 mg) and Bucinnazine, IM (*n* = 20) q8h	Responder rate (%)	80%	80%
Shen 2009 [18]	52 (23/25) Various	Stauntoniae injection at ear acupoints (*n* = 23) 0.1–0.3 mL at each point, qd	Oxycodon, po (*n* = 25) (1) moderate pain: 5 mg, bid (2) severe pain: 10 mg, bid (dose escalation by 25–50% if pain gets worse)	(A) Responder rate (%) (B) Onset of analgesia, duration of analgesia (min, mean ± SD)	(A) 91.3% (B) 17.7 ± 6.9**, 18.2 ± 1.3**	(A) 92% (B) 31.4 ± 6.6, 11.8 ± 1.0
Dou et al. 2004 [19]	120 (67/43) Late gastric cancer	Pethidine (50–100 mg) injection at ST36 (*n* = 43)	(1) MA at ST36 (*n* = 39) (2) Pethidine (50–100 mg), IM (*n* = 39)	Responder rate (%)	93%**	(1) 71.8% (2) 60.5%
Wang et al. 2004a [20]	102 (35/67) Various	Anisodamine (10 mg), dexamethasone (5 mg), and energy synbiotics injection (4 mL) at bilateral ST36, and Vit. B12 (500 *μ*g) at bilateral SP10 (*n* = 52) qd	Pethidine (100 mg), IM (*n* = 50) qd	Responder rate (%)^b^	0 h: 57.7%** 0.5–4 h: 80.8% 4–24 h: 75%** 24–72 h: 78.9%** 96 h: 80.8%**	0 h: 0% 0.5–4 h: 88% 4–24 h: 50% 24–72 h: 50% 96 h: 52%
Wang et al. 2004b [21]	120 (61/59) Various	Anisodamine (10 mg), dexamethasone (5 mg), and energy synbiotics injection (4 mL) at bilateral ST36, and Vit. B12 (500 *μ*g) at bilateral SP10 (*n* = 60) qd	Pethidine (100 mg), IM (*n* = 60) qd	Responder rate (%)^b^	0 h: 51.7%** 4 h: 93.3% 24 h: 81.7%** 72 h: 85%** 96 h: 91.7%**	0 h: 0% 4 h: 95% 24 h: 15% 72 h: 60% 96 h: 63.3%
Bai 2003 [22]	120 (71/49) Various	Nefopam injection (20 mg) at bilateral ST36 (*n* = 60)	Pethidine (50 mg), IM (*n* = 60)	Responder rate (%)	81.6%*	65%
Guan et al. 2001 [23]	122 (86/36) Various	Distilled and purified Chinese herbs (4 mL) injection at various acupoints (*n* = 61) qd (bid, if pain is severe)	Western medicine (4 mL) injection at various acupoints (*n* = 61) qd (bid, if pain is severe)	Responder rate (%)^b^	57.4%*	42.6%

AE: adverse events; bid: twice a day; h: hour(s); IM: intramuscular injection; MA: manual acupuncture; min: minute(s); NR: not reported; po: orally; q8h: every 8 hours; qd: everyday; SD: standard deviation; Vit.: vitamin.

Acupuncture point ST36 refers to 36th point of stomach meridian and extra points have different nomenclature (e.g., Ex-UE7 means 7th extra point in the upper extremity).

Responder rate: (number of participants with any improvement from the intervention/total number of participants) ∗ 100(%) unless stated otherwise.

^a^Responder rate: (number of participants with no need of analgesia/total number of participants) ∗ 100(%).

^b^Responder rate: (number of participants with improvement from the intervention with no sleep disturbance/total number of participants) ∗ 100(%).

*P < 0.05; **P < 0.01.

**Table 2:** Characteristics of the included studies: chemotherapy-induced nausea and vomiting (CINV).

Author	Sample size (M/F) Cancer type	Intervention (*n*)	Control (*n*)	Outcome measures	Result	
					Intervention	Control
Liu et al. 2011 [24]	51 (27/24) Hematologic cancer	Promethazine (25 mg) injection at bilateral ST36 (*n* = 26)	Metoclopramide (10 mg), IV (*n* = 25)	Responder rate (%)^a^	96.2%*	68%
Yang et al. 2011 [25]	96 (50/46) Various	Metoclopramide (10 mg) injection at bilateral ST36 (*n* = 50) 0.5 h before chemotherapy, qd	Tropisetron (5 mg), IV (*n* = 46) 0.5 h before chemotherapy, bid	Responder rate (%)^b^	78%*	52.1%
You et al. 2009 [26]	142 (0/142) Ovarian cancer	Vit. B6 (50 mg) injection and MA at bilateral PC6 (*n* = 46) qod	(1) Vit. B6 (50 mg), IM (*n* = 46) bid (2) MA at bilateral PC6 (*n* = 45) qod	(A) Total number of emesis episodes in 21 days (mean, 95% CI) (B) Proportion of emesis free days in 21 days (%, mean, 95% CI)	(A) 5.9, 3.8–7.0** (B) 59%, 48–69**	(1) (A) 13.2, 9.4–15.0 (B) 21%, 12–28 (2) (A) 10.6, 7.4–11.8 (B) 30%, 21–39
Chen 2007 [27]	258 (0/258) Ovarian cancer	Metoclopramide injection (*n* = 98) 10 mg, at left PC6 and CV10, 15 min before chemotherapy 10 mg, at right PC6 and CV 10 after chemotherapy	(1) Metoclopramide, IV (*n* = 74) 20 mg each, before and after chemotherapy (2) Granisetron (3 mg), IV (*n* = 86) before chemotherapy	Responder rate (%)^c^	95%**	(1) 40.5% (2) 96.5%
Hu 2003 [28]	123 (86/37) Various	Huangqi or Danggui (4 mL) injection, alternately at bilateral ST36, SP10, and BL23 (*n* = 71) qd	Batilol (50 mg), leucogen (20 mg) and Vit. B6 (20 mg), po (*n* = 52) tid	Responder rate (%)^b^	71.8%**	44.2%
Tao et al. 2000 [29]	480 (343/137) Various	Metoclopramide (5 mg) and diazepam (1.25 mg) injection alternately at bilateral PC6 and ST36 (*n* = 160) 30 min before chemotherapy Metoclopramide (10 mg) after chemotherapy	(1) Metoclopramide (20 mg) and diazepam (5 mg), IV (*n* = 160) 30 min before chemotherapy Metoclopramide (20 mg) after chemotherapy (2) Ondansetron, IV (*n* = 160) 8 mg, 30 min before chemotherapy 8 mg, after chemotherapy	Responder rate (%)^d^	98.8%**	(1) 43.1% (2) 94.4%

AEs: adverse events; bid: twice a day; CI: confidence interval; h: hour(s); IM: intramuscular injection; IV: intravenous injection; MA: manual acupuncture; min: minute(s); NR: not reported; po: orally; qd: everyday; qod: every other day; Vit.: vitamin; WHO: World Health Organization.

Acupuncture point ST36 refers to 36th point of stomach meridian and extra points have different nomenclature (e.g., Ex-UE7 means 7th extra point in upper extremity).

^a^Responder rate: (number of participants with any improvement from the intervention/total number of participants) ∗ 100(%).

^b^Responder rate: (number of participants with WHO grade [30] 0 + 1/total number of participants) ∗ 100(%).

^c^Responder rate: (number of participants with less than four emesis episodes per day/total number of participants) ∗ 100(%).

^d^Responder rate: (number of participants with less than three emesis episodes per day/total number of participants)∗100 (%).

*P < 0.05; **P < 0.01.

**Table 3:** Characteristics of the Included Studies: Ileus, Hiccup, Fever, QOL, Gastrointestinal Symptoms.

Author	Sample Size (M/F) Cancer Type	Intervention (*n*)	Control (*n*)	Outcome Measures	Result	
					Intervention	Control
*Ileus *						
Chen et al. 2010 [31]	120 (96/24) Rectal cancer	Neostigmine injection (0.5 mL) at bilateral ST36 (*n* = 60) bid Routine post-operative therapy	Routine post-operative therapy (*n* = 60)	Time that the patient first passed a bowel movement (h, mean ± SD)	45.1 ± 8.6*	74.7 ± 16.3
Feng et al. 2007 [32]	160 (92/68) Colon cancer	Vit. B1 injection (50 mg) at bilateral ST36 (*n* = 80) on the day of surgery	Vit. B1 (100 mg), IM (*n* = 80) on the day of surgery	Time that the patient first passed a bowel movement (h, mean ± SD)	29.6 ± 3.2	48.1 ± 5.3

*Hiccup *						
Sui and Zhang 2009 [33]	47 (31/16) Various	Anisodamine injection (10 mg) at ST36 (*n* = 25) bid	Anisodamine (10 mg), IM (*n* = 22) bid	Responder rate (%)	76%*	36.4%
Xia et al. 2000 [34]	32 (20/12) Various	Atropine injection (1.25 mg) at bilateral ST36, Vit. B1 (10 mg), B12 (10 mg) at bilateral BL17 (*n* = 16)	Atropine po (0.3 mg), tid or IM (0.5 mg) (*n* = 16)	Responder rate (%)	93.8%	68.8%

*Fever *						
Yan et al. 1999 [35]	28 (21/7) Various	rIL-2 injection (50,000 *μ*/2 mL) at ST36 (*n* = 14) qd	rIL-2 (100,000 *μ*/2 mL), IM (*n* = 14) qd	Responder rate (%)	85.7%*	50%
Yan et al. 1997 [36]	58 (43/15) Upper GI cancer	Dexamethasone injection (1 mL) alternately at bilateral ST36 (*n* = 28) qd	Dexamethasone (10 mg), IM (*n* = 30) tid	Responder rate (%)	89.3%**	56.7%

*QOL *						
Xue 2005 [37]	108 (78/30) Various	Huangqi injection (2 mL) alternately at bilateral ST36 (*n* = 60) qd	Energy synbiotics, IV (*n* = 48) qd Lipid and albumin, twice a week	Responder rate (%, Karnofsky score)	50%**	25%

*GI Symptoms *						
Zhao et al. 2008 [38]	30 (11/19) Various	Metoclopramide injection (10 mg) at bilateral ST36 (*n* = 15) qd Routine chemotherapy	Metoclopramide (10 mg), IM (*n* = 15) qd Routine chemotherapy	WHO grade [30] 0, 1, 2, 3, 4 (number of patients)	Grade 0: 1 Grade 1: 6 Grade 2: 8 Grade 3: 0 Grade 4: 0	Grade 0: 0 Grade 1: 4 Grade 2: 9 Grade 3: 2 Grade 4: 0

Abbreviation: AEs: adverse events; bid: twice a day; GI: gastrointestinal; h: hour(s); IM: intramuscular injection; IV: intravenous injection; min: minute(s); NR: not reported; po: orally; qd: everyday; QOL: quality of life; rIL-2: recombinant human interleukin-2; SD: standard deviation; tid: three times a day; Vit.: vitamin; WHO: World Health Organization.

Acupuncture point ST36 refers to 36th point of stomach meridian and extra points have different nomenclature (e.g., Ex-UE7 means 7th extra point in upper extremity).

Responder rate: (number of participants with any improvement from the intervention/total number of participants) ∗ 100(%) unless stated otherwise.

*P < 0.05; **P < 0.01.

**Table 4:** ROB assessment for the included studies by symptom: pain, nausea and vomiting, ileus, hiccup, fever, QOL, and gastrointestinal symptoms.

Author	Random sequence generation	Allocation concealment	Blinding of participants	Blinding of outcome assessment	Incomplete outcome data	Selective reporting
Pain						
Liu 2010 [16]	U	U	N	N	Y	Y
Li 2009 [17]	U	U	N	N	Y	Y
Shen 2009 [18]	U	U	N	N	Y	Y
Dou et al. 2004 [19]	U	U	N	N	Y	Y
Wang et al. 2004a [20]	U	U	N	N	Y	Y
Wang et al. 2004b [21]	U	U	N	N	Y	Y
Bai 2003 [22]	U	U	N	N	Y	Y
Guan et al. 2001 [23]	U	U	N	N	Y	Y

Nausea and vomiting						
Liu et al. 2011 [24]	U	U	N	N	Y	Y
Yang et al. 2011 [25]	U	U	N	N	Y	Y
You et al. 2009 [26]	U	Y	N	N	Y	Y
Chen 2007 [27]	U	U	N	N	Y	Y
Hu 2003 [28]	U	U	N	N	Y	Y
Tao et al. 2000 [29]	U	U	N	N	Y	Y

Ileus						
Chen et al. 2010 [31]	U	U	N	N	Y	Y
Feng et al. 2007 [32]	U	U	N	N	Y	Y

Hiccup						
Sui and Zhang 2009 [33]	U	U	N	N	Y	Y
Xia et al. 2000 [34]	U	U	N	N	Y	Y

Fever						
Yan et al. 1999 [35]	U	U	N	N	Y	Y
Yan et al. 1997 [36]	U	U	N	N	Y	N

QOL						
Xue 2005 [37]	U	U	N	N	Y	Y

Gastrointestinal symptoms						
Zhao et al. 2008 [38]	U	U	N	N	Y	Y

ROB was assessed using the ROB assessment tool from the Cochrane Handbook for Systematic Reviews of Interventions [39].

N: no (high risk of bias); QOL: quality of life; ROB: risk of bias; U: Unclear (uncertain risk of bias); Y: yes (low risk of bias).

**Table 5:** Reported AEs in the included studies.

Author Sample Size* (Int./Cont.)	Condition	Intervention group	Control group
Reported “No AEs” for intervention group			
Liu et al. 2011 [24]	CINV	No AEs occurred	No AEs occurred
Yang et al. 2011 [25]	CINV	No AEs occurred	NR
Chen 2007 [27]	CINV	No AEs occurred	NR
Xue 2005 [37]	QOL	No AEs occurred	NR
Shen 2009 [18] 23/25	Pain	No AEs occurred	Constipation (9), nausea and vomiting (6), dizziness (3)

Identical Drug Used for Both Groups			
Liu 2010 [16] 28/19	Pain	Nausea and vomiting (1), headache and dizziness (2) Resolved after the end of treatment	Nausea and vomiting (3), headache and dizziness (5), drowsiness and fatigue (2) Resolved after the end of treatment
Sui and Zhang 2009 [33] 25/22	Hiccup	Xerostomia (9) Resolved after the end of treatment	Xerostomia (10) Resolved after the end of treatment
Tao et al. 2000 [29]** 160/160	CINV	Headache and dizziness (26), bloating and constipation (4), and diarrhea (6) Resolved after the end of treatment	Headache and dizziness (33), bloating and constipation (9), and diarrhea (20) Resolved after the end of treatment
Yan et al. 1997 [36] 28/30	Fever	Local pain and limitation of lower limb movement (5) Resolved after the end of treatment	Insomnia and increased talkativeness (8) and panhidrosis (2) Did not affect further treatment

Different Drugs Used for Each Groups			
Wang et al. 2004a*** [20]	Pain	Xerostomia, hot flashes, and blurred vision Relieved spontaneously	Nausea, vomiting, constipation, and urinary retention Mild and tolerable level
Wang et al. 2004b*** [21]	Pain	Xerostomia, hot flashes, and blurred vision Relieved spontaneously Temporary low blood sugar Continued treatment after glucose IV injection or a meal	Nausea, vomiting, constipation, and urinary retention Mild and tolerable level
Tao et al. 2000 [29]** 160/160	CINV	Headache and dizziness (26), bloating and constipation (4), and diarrhea (6) Resolved after the end of treatment	Headache and dizziness (29), bloating and constipation (22), and diarrhea (5) Resolved after the end of treatment

AEs: adverse events; CINV: chemotherapy-induced nausea and vomiting; Cont.: control group; Int.: intervention group; IV: intravenous; NR: not reported; QOL: quality of life.

Number in the parenthesis is reported number of cases.

*Sample size is reported on this table for studies with numerical data only.

**Two different control groups yielded two separate documentations in this table.

***Number of cases for symptoms were not reported.
